# Exposure to metoprolol and propranolol mixtures on biochemical, immunohistochemical, and molecular alterations in the American oyster, *Crassostrea virginica*

**DOI:** 10.1016/j.toxrep.2025.101979

**Published:** 2025-03-04

**Authors:** Andrew Salinas, Md Saydur Rahman

**Affiliations:** a School of Integrative Biological and Chemical Sciences, University of Texas Rio Grande Valley, Brownsville, TX, USA; b School of Earth, Environmental, and Marine Sciences, University of Texas Rio Grande Valley, Brownsville, TX, USA

**Keywords:** Pharmaceuticals, Beta-blockers, Aquatic environment, Nitrative stress, AChE, Marine bivalves

## Abstract

Pharmaceutical drugs, particularly beta-blockers (e.g., metoprolol, propranolol, etc.), are extensively used to treat human cardiovascular conditions, yet pose significant risks to non-target aquatic organisms when introduced into coastal and marine environments via wastewater effluent. This study aimed to investigate the effects of short-term exposure (one week) to environmentally relevant concentrations of metoprolol and propranolol (MP) mixtures (low-dose: 50 ng/L propranolol and 250 ng/L metoprolol, and high-dose: 250 ng/L propranolol and 650 ng/L metoprolol) in the American oyster (*Crassostrea virginica*, a commercially and ecologically important marine bivalve mollusk) under controlled laboratory conditions. Histopathological assessments revealed structural damage to gills, connective tissues, and digestive glands in both low- and high-dose MP treatment groups. Additionally, glucose concentration and pH of the extrapallial fluid significantly declined in the high-dose MP treatment groups. Hemocyte density in the connective tissues increased proportionally with MP dosages. MP mixtures significantly reduced mucous secretion in the gills and digestive glands. Immunohistochemical results showed significant (*P* < 0.05) upregulation of 3-nitrotyrosine protein (NTP, a biomarker of protein nitration) expression in tissues of oysters exposed to MP mixtures. Alongside, exposure to MP significantly (*P* < 0.05) decreased acetylcholinesterase (AChE, a cholinergic enzyme) expression in oyster tissues. Our findings suggest that beta-blockers induce protein nitration, leading to altered tissue morphology, disrupting extrapallial fluid homeostasis, and downregulating AChE expression that may impair physiological functions in oysters.

## Highlights

• Aquatic environments are becoming increasingly inundated by pharmaceuticals.
• Pharmaceutical drugs harm non-target aquatic organisms.
• Beta-blockers altered the morphology of gills and digestive glands of oysters.
• Beta-blockers induced nitrative stress and downregulated AChE expression in oysters.

## Graphical Abstract

**Figure:**
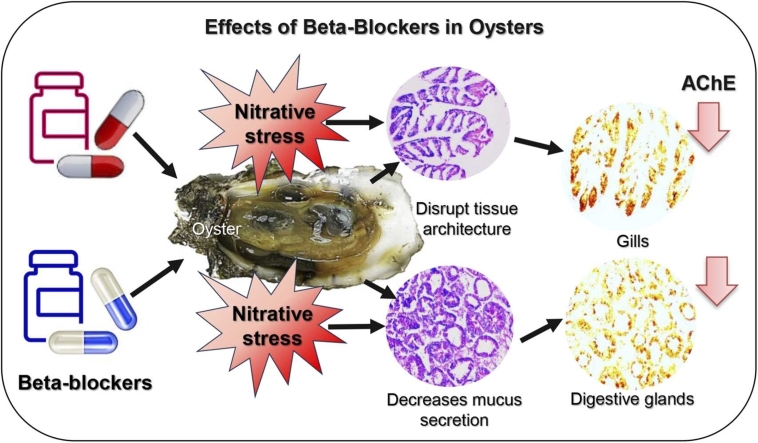

## 1 Introduction

The disposal of pharmaceutical substances presents a growing threat to ecosystems. Concerns have intensified regarding the effects of pharmaceutical contaminants on diverse environments, particularly marine and coastal waters, due to their increasing prevalence [1]. These substances can enter aquatic environments through various routes, such as the release of processed wastewater from sewage treatment facilities, improper disposal of leftover medications, and agricultural runoff containing pharmaceuticals used in livestock care [2], [3]. When introduced into aquatic ecosystems, these compounds can remain, build up, and interact with marine organisms, leading to diverse biological and ecological effects [4], [5], [6].

Extensive research has explored the occurrence of pharmaceuticals in marine environments and their effects on aquatic organisms [5], [7], [8], [9]. Some studies indicate that these substances can directly alter physiological processes in aquatic species [10], [11], [12], [13], [14], while others suggest indirect impacts by modifying food sources or changing the structure of ecosystems [15]. With detecting a wide range of pharmaceutical compounds in marine environments, understanding which specific drugs present the highest risks is essential [16]. Evidence suggests that certain pharmaceutical categories, such as antibiotics and medications used to manage hypertension, may have particularly severe effects on marine life [17], [18]. Identifying which pharmaceutical compounds are most harmful to specific species is critical for evaluating ecological risks and guiding conservation strategies [19]. Additionally, further research is needed to explore the long-term effects of pharmaceutical contamination impacts marine organisms and ecosystems, and long-term research could offer valuable insights into shifts in community composition, reproductive outcomes, or heightened vulnerability in regions with different contamination intensities [20]. Advancing knowledge in these areas will support the development of measures to reduce pharmaceutical impacts on marine ecosystems. As pharmaceutical usage rises, it is vital to implement strategies to protect marine habitats from contamination to preserve biodiversity and the health of marine and coastal organisms [20].

Understanding how environmental pharmaceuticals induce oxidative and nitrative stress is essential for comprehending their impact on cellular functions and organisms. The aim is to investigate how even minimal concentrations of these chemicals can trigger oxidative and nitrative stress, disrupting the balance between the production of reactive oxygen species (ROS) or reactive nitrogen species (RNS) and the body's antioxidant defenses [20]. Such imbalances can harm ecosystems and animal health. Research has revealed the intricate connection between environmental pharmaceuticals and oxidative/nitrative stress, detailing their mechanisms and consequences [21], [22], [23]. Various pharmaceutical substances, including antibiotics, analgesics, hormones, antidepressants, and anti-vaccines, have been detected in the environment [7], [24]. These compounds are found in surface water, groundwater, sediments, and biota. Exposure to these substances can trigger the production of ROS and RNS [20]. Elevated production of ROS/RNS, coupled with diminished antioxidant defenses, results in oxidative/nitrative stress, which can cause cellular damage, genetic mutations, and interference with essential biological functions [25]. For example, marine organisms such as clams (*Ruditapes philippinarum*), snails (*Gibbula umbilicalis*), and ragworms (*Hediste diversicolor*) have been shown to accumulate peroxidation products like lipofuscin and malondialdehyde, damage DNA, increase the frequency of micronuclei, decrease in neutral lipids, and inhibit acetyl coenzyme A (Acetyl-CoA) activity in tissues of organisms [26], [27], [28]. Research into how pharmaceuticals induce oxidative/nitrative stress is a multidisciplinary effort encompassing ecotoxicology, physiology, pharmacology, and environmental and marine sciences. Current literature underscores the need for a comprehensive evaluation of pharmaceutical effects on both individual species and ecosystem health [29]. By addressing these challenges, researchers can inform policy decisions, guide regulatory frameworks, and improve remediation strategies to minimize the adverse effects of pharmaceuticals on aquatic life through oxidative and nitrative stress [24].

Acetylcholinesterase (AChE) is an enzyme that is fundamental to cholinergic neurotransmission in diverse organisms, including marine bivalve mollusks [30], [31]. This enzyme plays a vital role in neurotransmission and neuroplasticity, which is essential for the proper functioning of the nervous system [32], [33]. Notably, AChE catalyzes the hydrolysis of acetylcholine to acetate and choline, which is necessary for terminating the synaptic transmission at cholinergic synapses and for orderly physiological functions [30]. In oysters, acetylcholine has a key role in muscle contraction and physiological processes [31]. Moreover, evidence has been accumulated that AChE plays a vital role in oyster muscle function that is important for feeding, respiration, and other critical physiological activities [34].

Beta-blockers such as metoprolol and propranolol are widely employed classes of pharmaceuticals used in the treatment of human cardiovascular conditions [35]. These drugs antagonize beta-adrenergic receptors in the sympathetic nervous system, that are activated by the neurotransmitters noradrenaline and adrenaline [36]. Beta-blockers and other pharmaceuticals end up in the aquatic environment because of pharmaceutical runoff, where they can act on non-target organisms such as fish, shellfish, and aquatic invertebrates [37]. Since beta-blockers can interact with neurotransmitter systems, therefore, their effects on aquatic organisms are of particular concern, as it has been found that beta-blockers can induce oxidative/nitrative stress and indirectly affect AChE activity, by modulating sympathetic neurotransmitter systems and thereby changing cholinergic pathways, such as the release and breakdown of acetylcholine by AChE [27]. Moreover, beta-adrenergic and cholinergic enzymes in oysters are connected through a complex feedback game, such that manipulating activity in one system using a beta-blocker ultimately has secondary effects on all other physiological effects [37], [38]. If beta-blockers truly affect muscle strength and energy generation (i.e., metabolites) in oysters, hence, we have an important new tool for understanding how pharmaceutical drugs like beta-blockers can impact aquatic organisms. Therefore, it is hypothesized that exposure to the beta-blocker mixture of metoprolol and propranolol will negatively alter the morphology of tissues in oysters. In conjunction with morphological damage, we expect to observe an impact on body fluid conditions, nitrative stress biomarkers, and AChE expression in the tissues of oysters exposed to beta-blockers.

Using the American oyster (*Crassostrea virginica,* an edible and commercially cultivated shellfish species in the United States and Mexico) as a model marine mollusk, three main objectives were pursued: (i) to investigate the effects of metoprolol and propranolol (MP) mixtures on tissue architectures and mucus secretion in the gills and digestive glands of American oysters, (ii) to examine the effects of MP mixtures on extrapallial fluid (also called body fluid) conditions in oysters, and (iii) to explore the potential mechanisms on 3-nitrotyrosine protein (NTP, a biomarker of protein nitration) and AChE regulation in the tissues of oysters.

## 2 Materials and methods

### 2.1 Experimental oysters

American oysters (average age: 2–3 years*)* were collected during low tide from seaside in the Gulf of Mexico coast in South Padre Island, Texas. All oysters were collected from adjacent to human recreational activities such as jet skiing and recreational fishing. The sampling sites of oysters are free from point sources of marine pollutants like sewage treatment plants, environmental chemicals, etc. [39]. Oysters were kept in buckets with aerated seawater and transported to the laboratory at the University of Texas at Rio Grande Valley in Brownsville campus. To remove any remaining clinging debris or persistent fouling organisms, oysters were subsequently rinsed with filtered seawater and placed in six glass aquariums (12–15 oysters/aquarium, 75-L capacity; Tetra, Blacksburg, VA, USA). Before exposure to beta-blockers (i.e., propranolol and metoprolol mixture), oysters underwent a 1-week acclimatization period with recirculating aerated seawater under controlled room temperature (22 ± 0.5 °C) and constant light-dark (12:12) cycle. To maintain water quality in aquariums, the seawater was changed every other day at a ratio of 25 % per change. This ensured that ammonia and nitrite levels did not surpass 0.1 ppm in aquariums.

### 2.2 Experimental protocols and tissue collection

After acclimatization, two aquariums were designated as controls and other two aquariums were randomly selected to receive a low dose of propranolol (50 ng/L) (purity >99 %, CAS-No: 318–98–9, MilliporeSigma, Burlington, MA, USA) and metoprolol (250 ng/L) (purity >99 %, CAS-No: 56392-17-1, MilliporeSigma) mixture, and the remaining two aquariums were randomly selected to receive a high dose of propranolol (250 ng/L) and metoprolol (650 ng/L) mixture. The concentrations of metoprolol and propranolol used in this study are based on environmental concentrations (metoprolol: 237–660 ng/L, propranolol: 53–260 ng/L [40], [41]). Experimental pharmaceutical levels were maintained by removing activated carbon filtration from each aquarium before the first dosage and redosing in conjunction with water changes. Water quality such as dissolved oxygen levels, pH, and temperature were measured three times daily throughout the experiment using a YSI probe (YSI Professional Plus 1020 Multiprobe System, Yellow Springs, OH, USA). The oysters were fed frozen marine cuisine (crude protein: 7.1 %, fat: 1.7 %, and fiber 0.6 %; San Francisco Bay Brand, Inc.) every alternating day during the experimental period.

After one week of exposure to metoprolol and propranolol mixtures, ten oysters were collected from each aquarium. Oysters were carefully shucked to prevent extrapallial fluid spillage. Extrapallial fluid was collected immediately, pH was measured with a portable digital pH meter (Eutech Instruments, Waltham, MA, USA), and stored in 1.5-mL tubes at −80°C for biochemical analysis. Gills and digestive glands tissue samples were carefully extracted, placed into respective histology cassettes, immersed in 4 % ice-cold paraformaldehyde solution (Acors Organics, Morris, NJ, USA), and stored at 4°C for histological and immunohistological analyses. Tissue samples were also collected in RNase-DNase-free tubes, quickly frozen on dry ice, and stored at −80°C for molecular analysis.

### 2.3 Histological analysis in tissues

After fixation with paraformaldehyde, tissue samples underwent dehydration using a gradient of ethanol solutions (50 %, 75 %, 95 %, and 100 %). Each step involved immersing the tissues in glass vials containing the respective ethanol concentration for 30 min, with two immersions in 100 % ethanol. Following dehydration, tissue samples were then treated twice with xylene for 15–30 min each time. Tissue samples were then cleansed with melted paraffin (Fisher Scientific, Hampton, NH, USA) and embedded using molds and cassettes. Embedded tissues were sectioned at a thickness of 5 μm using a rotary microtome machine (Lecia, Weltzar, Germany) and mounted on positively charged glass slides (SuperfrostTM Plus®, Thermo Fisher, Waltham, MA, USA). After drying on a warmer plate, the slides were stored at −80 °C until histological and immunohistochemical analyses. For histological analysis, slides were deparaffinized with xylene, rehydrated with a series of decreasing ethanol concentrations (100 %, 95 %, 75 %, and 50 %), and then stained with hematoxylin (MilliporeSigma, St. Louis, MI, USA) and eosin (Fisher Scientific) solutions following standard protocols described by Dash and Rahman [39]. Stained sections were observed under a light trinocular compound microscope and images were captured using a 5MP digital camera (AmScope, Irvine, CA, USA).

### 2.4 Periodic acid-Schiff staining in tissues

Periodic acid-Schiff (PAS) reaction was used to test the mucus secretion in the gills and digestive glands according to the protocol described by Ahmed and Rahman [42]. Briefly, tissue sections were deparaffinized using xylene and rehydrated through a series of decreasing ethanol concentrations (100 %, 95 %, 75 %, and 50 %). Slides were then oxidized in 0.5 % periodic acid solution for five min, followed by rinsing in distilled water for 5 min 3 times each. To stain the tissue sections, slides were immersed in Schiff's reagent for 15 min, resulting in a light pink color. Slides were then rinsed in tap water for 5 min, changing the color of the tissue sections to dark pink. Sections were counterstained using hematoxylin solution for 1 min, followed by rinsing in tap water for 5 min and then washed in distilled water. Finally, slides were dehydrated using a gradient of ethanol solutions and mounted with coverslips using a xylene-based mounting media. Stained sections were examined under a light microscope and captured images using a digital camera (AmScope).

### 2.5 Glucose analysis in extrapallial fluid

Extrapallial fluid glucose concentration was determined using a HemoCue glucose 201 analyzer following the manufacturer's protocol (Angel Holm, Sweden).

### 2.6 Immunohistochemical analysis in tissues

Immunohistochemical analysis was assessed to measure protein expressions in the gills and digestive glands following the method outlined by Nash and Rahman [43]. Briefly, tissue sections were mounted on slides, deparaffinized using xylene, and rehydrated with a series of ethanol solutions. The slides were then washed three times in 1x phosphate-buffered saline (PBS, Fisher Scientific) and incubated with 1 % bovine serum albumin to block non-specific protein binding for 1 h at room temperature. The slides were then rinsed again in 1x PBS. The primary antibodies, mouse anti-NTP (Santa Cruz Biotechnology, Dallas, TX, USA) or rabbit anti-AChE (Novus Biological, Centennial, CO, USA), were diluted 1:100 in 1x PBS and applied to the slides. Both antibodies were validated previously in oyster tissues according to the method described by Dash and Rahman [39]. Briefly, the negative control slides were incubated with PBS instead of anti-NTP and anti-AChE antibodies. The slides were then incubated for 48 h at 4°C. After washing with 1x PBS, the slides were then incubated with anti-mouse (Cell Signaling, Denver, MA, USA) or anti-rabbit (Southern Biotech, Birmingham, AL, USA) secondary antibody (1:100 in 1x PBS) for 2 h at room temperature. Following this, the slides were washed three times with 1x PBS (15 min each). To detect NTP or AChE expression in the gill and digestive gland tissues, 3,3-diaminobenzidine (DAB) peroxidase substrate (Vector Laboratories, Newark, CA, USA) was applied in the dark. The slides were then washed with deionized water and xylene, dehydrated through a graded ethanol dilution, and mounted with Cytoseal glue (XYL 60, Thermo Fisher). The immunoreactive (IR) intensities of NTP or AChE were visually detected in a light microscope and images were photographed using a digital camera (AmScope). The IR intensity was measured by the optical density (OD) of the images using ImageJ software following the methods of Schneider et al. [44] and Rahman and Rahman [45].

### 2.7 Quantitative real-time PCR analysis in tissues

Total RNA was extracted from gill and digestive gland tissue samples using TRI-reagent according to the manufacturer protocol (MilliporeSigma). RNA concentration was measured by NanoDrop and integrity was verified using agarose gel electrophoresis. The mRNA expression levels were quantified by real-time PCR using the One-Step SYBR Green-qRT-PCR master mix (Promega, Madison, USA) according to the method described previously by Dash and Rahman [39]. The mRNA expression levels of AChE were calculated using the 2^-ΔΔCt^ method [46] and normalized to levels of oyster elongation factor (EF)-1α gene expression. The primer sequences of AChE and EF-1α genes are shown in Table 1.

**Table 1:** Primer sequences used in quantitative real-time PCR for mRNA levels analyses in this study.

Candidate gene	Length (bp)	Accession no	Sense primer (5’ - 3’)	Anti-sense primer (5’ - 3’)
AChE	236	XM_022460884	AGAGCACCCAAGAACCTCCT	CCGACTCAGCCTGAATAAGC
EF−1α	200	JX117894	AGGCTGACTGTGCTGTGTTG	TTCAGCCTTGATTTCGTTGA

AChE, acetylcholinesterase; EF-1α, elongation factor-1α.

### 2.8 Statistical analysis

To compare the groups, a one-way analysis of variance (ANOVA) was used. Significant differences were performed using Tukey’s test for multiple comparisons and the Student’s *t*-test for unpaired comparison. A *P* value of < 0.05 was considered to be statistically significant throughout the analysis. Replicated results were combined to ensure statistically justified aggregation. Outliers were excluded to mitigate undue influence on group comparisons. All data were also checked for homogeneity. All analyses were conducted using GraphPad Prism software (GraphPad, San Diego, CA, USA).

## 3 Results

### 3.1 Effects of metoprolol and propranolol mixtures on tissue architecture

**Fig. 1:**
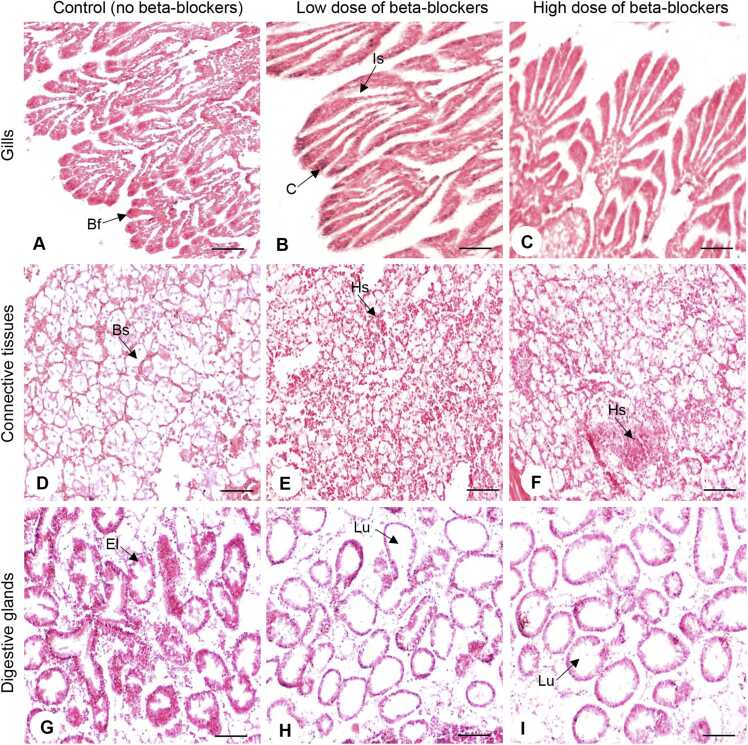
Effects of beta-blockers on tissue architectures of American oysters. Representative photographs of gill (A–C), connective tissue (D–F), and digestive gland (G–I) sections (hematoxylin-eosin staining) collected from oysters exposed to control (A, D, G), low dose (B, E, H), and high dose (C, F, I) of metoprolol and propranolol mixtures for one week. Scale bar = 100 µm. Bf, branchial filament; Bs, blood sinus; C, cilia; El, epithelial lining; Hm, hemocyte; Isa, intra-lamellar.

Our study showed notable morphological and histological alterations in oysters exposed (one week) to metoprolol and propranolol (MP) mixtures (Fig. 1). Oysters in the control group, not introduced to MP mixtures, displayed well-characteristic tissue structures such as even ciliated epithelial cells and intact branchial filaments in the gills (Fig. 1A), alongside uniformly structured in the connective tissues (Fig. 1D) and lumens of digestive glands (Fig. 1G). On the other hand, short-term exposure (i.e., one week) to MP mixtures (low dose: metoprolol: 250 ng/L and propranolol 50 ng/L; and high dose: metoprolol: 650 ng/L and propranolol: 250 ng/L) triggered a variety of effects in the gills (Fig. 1B, C), connective tissues, (Fig. 1E, F) and digestive glands (Fig. 1H, I).

**Fig. 2:**
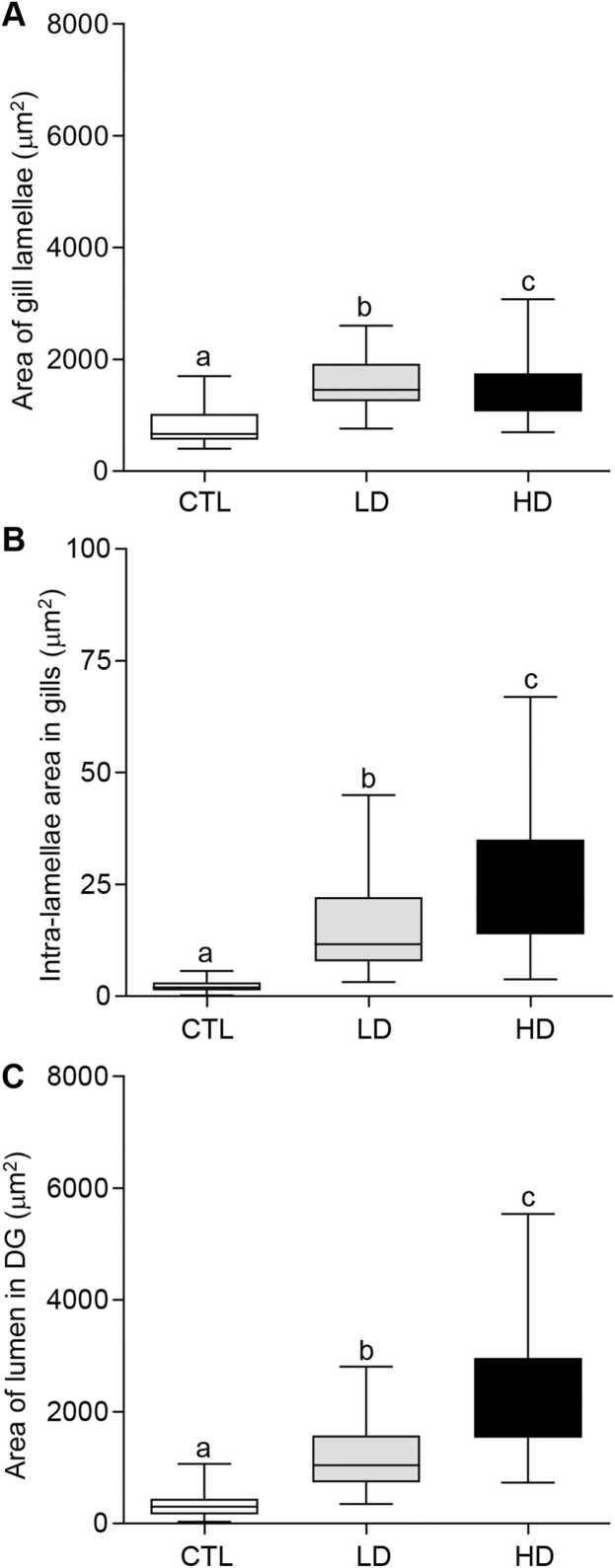
Effects of beta-blockers on tissue morphology of American oysters. Short-term (one week) exposure to metoprolol and propranolol mixtures on the area of gill lamellae (A), intra-lamellar area in gills (B), and area of lumen in digestive glands (DG) in oysters. Whiskers indicate the minimum and maximum values for each data set. The center line within the whisker box indicates the mean value (N = 55–83). Different letters indicate significant differences (one-way ANOVA followed by Tukey’s test, *P* < 0.05). CTL, control; LD, low dose; HD, high dose.

In the gill tissues, deterioration was observed in oysters exposed to MP mixtures (Fig. 1B, C). Damaged branchial filaments were observed in the gills. The area of gill lamellae increased significantly (*P* < 0.05, Tukey’s test) in both the low- and high-dose of MP mixture groups (Fig. 2A). The average area of the gill lamellae in the control group was (808.9 ± 43.64 µm), which was around 1.94-fold lower than the low-dose (1571 ± 50.58 µm) and ∼1.83-fold lower than the high-dose (1478 ± 74.54 µm) MP treatment groups (Fig. 2A). The intra-lamellar space in gill tissues was also measured and results displayed evidence of deterioration and thinning of gills (Fig. 1A–C). The control group displayed healthy-looking gills and had consistent space within the gill lamellae (Fig. 1A). The intra-lamellar space expanded and became more abundant with increasing dosages, from control to low-dose, and then to high-dose MP treatment groups (Fig. 1B, C). Exposure to MP mixtures caused the intra-lamellar spaces to become uneven and significantly enlarge (*P* < 0.05) as dosages increased (Fig. 2B). The average intra-lamellar area in the control group's gill tissue was (2.25 ± 0.14 µm). The low dose of the MP treatment group displayed an average intra-lamellar area of (15.87 ± 1.14 µm), around 7-fold higher than the control group. Furthermore, the high-dose of the MP group exhibited an average of 24.64 ± 1.77 µm, making it ∼11-fold larger than the low-dose treatment group (Fig. 2B). In the digestive glands, the epithelial lining within the lumen decreased in size as the dosage increased in oysters exposed to MP mixtures (Fig. 1H, I). When measuring the circumference of the lumen of digestive glands, the enlargement of the lumen became increasingly noticeable (*P* < 0.05, Tukey’s test) with both low and high- dosages of MP treatment groups (Fig. 2C). The average area of the lumen in the control group was (331.1 ± 22.68 µm). The low-dose MP exposure group exhibited an average lumen area of 1163 ± 58.08 µm, which was around 3.5-fold higher than the control group. The high-dose MP exposure group showed an average lumen area of 2371 ± 114 µm, making it ∼7.2-fold larger compared to the control group (Fig. 2C).

### 3.2 Effects of MP mixtures on mucous secretion in tissues

**Fig. 3:**
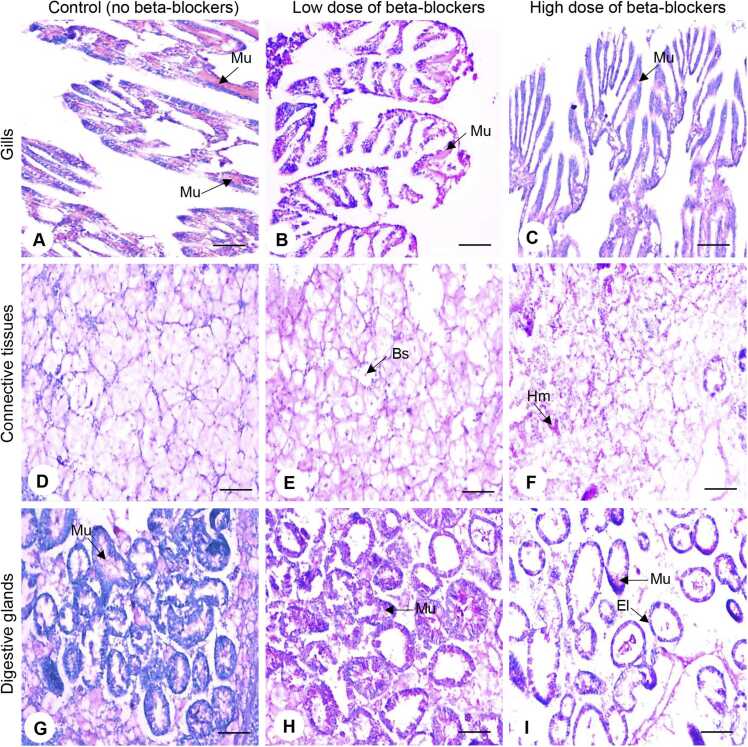
Effects of beta-blockers on tissues of American oysters. Representative photographs of gill (A–C), connective tissue (D–F), and digestive gland (G–I) sections (PAS staining) collected from oysters exposed to control (A, D, G), low dose (B, E, H), and high dose (C, F, I) of metoprolol and propranolol mixtures for one week. Scale bar = 100 µm. Bf, branchial filament; El, epithelial lining; Hm, hemocyte; Mu, mucus.

**Fig. 4:**
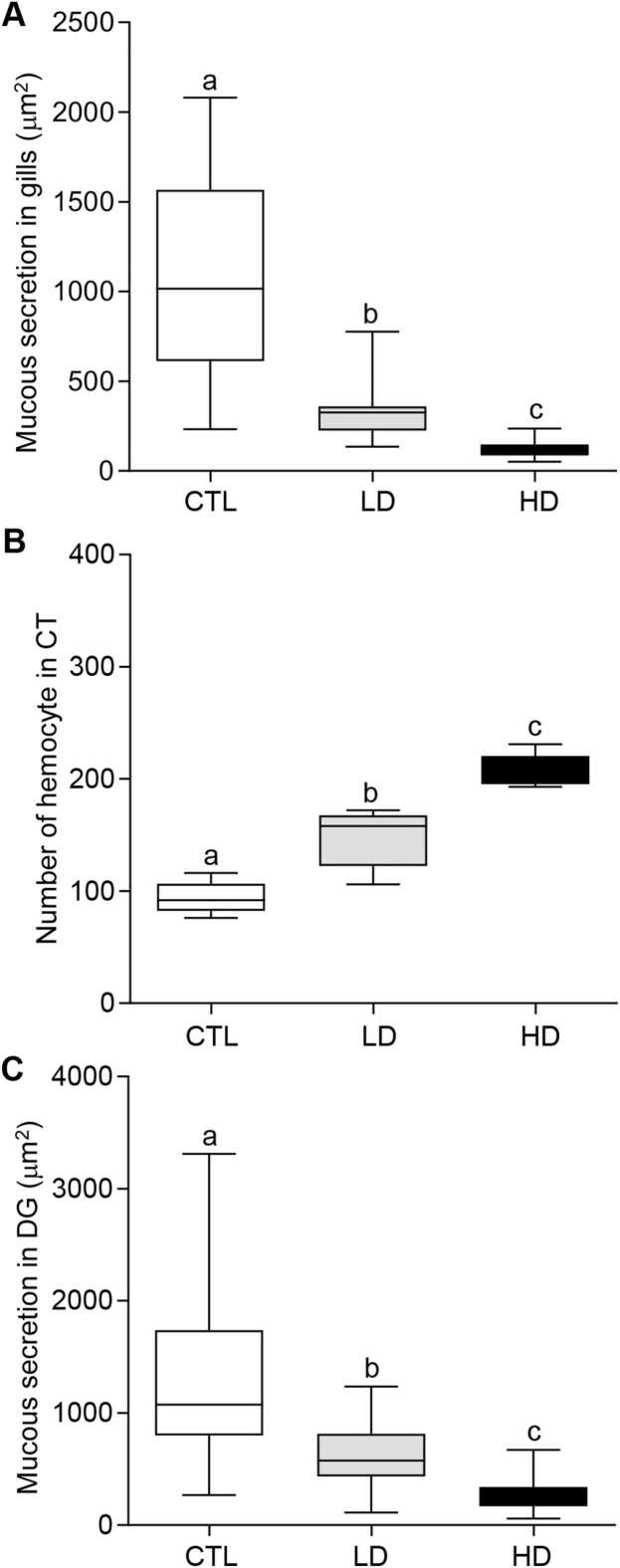
Effects of beta-blockers on tissue morphology of American oysters. Short-term (one week) exposure to metoprolol and propranolol mixtures on the area of mucus secretion in gills (A), number of hemocyte in connective tissues (CT) (B), and mucus secretion in digestive glands (DG) in oysters. Whiskers indicate the minimum and maximum values for each data set. The center line within the whisker box indicates the mean value (N = 41–61 for Fig. A, C; N = 5 for Fig. B). Different letters indicate significant differences (one-way ANOVA followed by Tukey’s test, *P* < 0.05). CTL, control; LD, low dose; HD, high dose.

To assess the secretion of mucus in the gills and digestive glands of oysters exposed to MP treatment groups, periodic acid-Schiff (PAS) staining was performed (Fig. 3). Mucous secretion was drastically depleted in the gills of oysters exposed to MP mixture groups (Fig. 3B, C). The mucous secretion significantly decreased (*P* < 0.05) in the gills of oysters exposed to both low- and high-dose MP treatment groups (Fig. 9). In the low- and high-dose treatment groups, the mucous secretion in the gills were ∼3.4- and ∼8.8-fold lower than the control group, respectively (Fig. 4A).

In the connective tissues, hemocytes were evenly dispersed (Fig. 3D). Small blood sinuses were present in the control group of oysters. As the MP dosages increased, the oysters exhibited hemocyte clustering, becoming more prevalent, and enlargement of the blood sinuses as an inflammatory reaction in the connective tissues (Fig. 3E, F). Counting of the hemocyte numbers showed that the MP mixtures directly affected an increase in hemocyte numbers within connective tissues (Fig. 4B). The average hemocyte count in the control group was 94 ± 6.5. The average hemocyte count was increased to 147.6 ± 11.72 in the low-dose MP treatment group, which was around 1.6-fold higher than the control group. The high-dose treatment group also showed an even greater increase, with an average hemocyte count of 207 ± 6.63, ∼2.2-fold higher than the control group (Fig. 4B).

Similar to the gills, mucus was also unevenly distributed and accumulated in the lumen of digestive glands in oysters exposed to MP mixtures (Fig. 3H, I). The quantity of mucous was significantly decreased (*P* < 0.05) in digestive glands in both low- and high-dose MP treatment groups (Fig. 4C). Mucus secretion was significantly reduced (*P* < 0.05) around 2.4-fold in low-dose and ∼4.7-fold in high-dose of MP treatment groups compared to the control groups (Fig. 4C).

### 3.3 Effects of MP mixtures on body fluid conditions

**Fig. 5:**
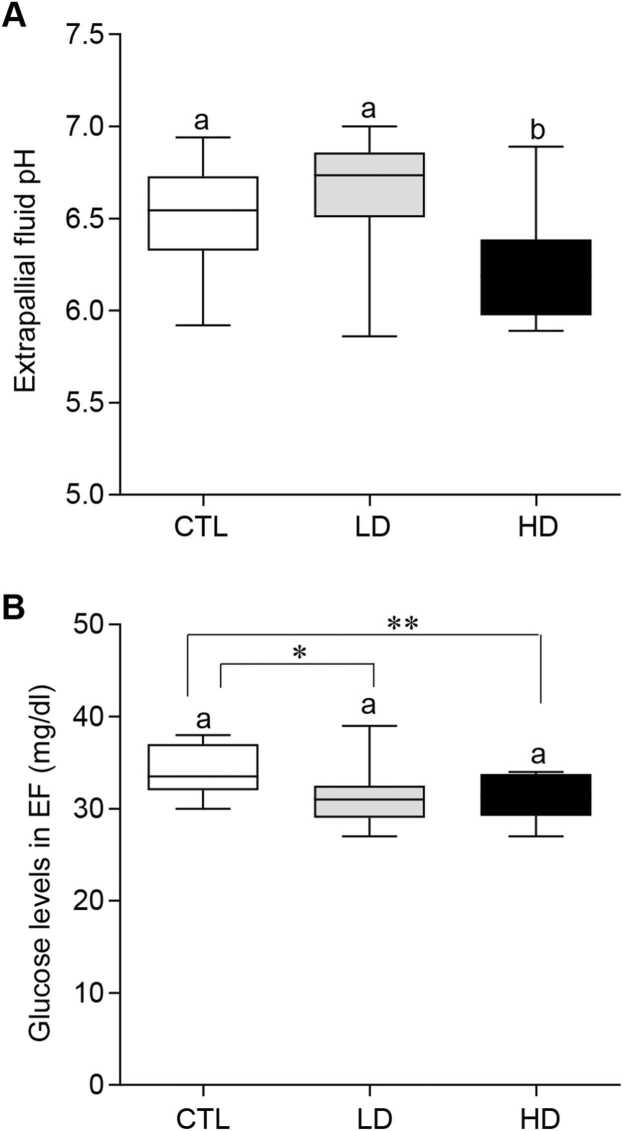
Effects of beta-blockers on extrapallial fluid conditions of American oysters. Short-term (one week) exposure to metoprolol and propranolol mixtures on extrapallial fluid (A) and glucose levels (B) in oysters. Whiskers indicate the minimum and maximum values for each data set. The center line within the whisker box indicates the mean value (N = 10–20). Different letters indicate significant differences (one-way ANOVA followed by Tukey’s test, *P* < 0.05). Asterisks indicate a significant difference (Student *t*-test). CTL, control; LD, low dose; HD, high dose.

The pH and glucose levels in oysters were measured to assess further the impact of MP on their body fluid (i.e., extrapallial fluid). The average pH of the control group oysters was 6.49 ± 0.06, while the low-dose treatment group averaged 6.64 ± 0.06, with no significant difference (*P* < 0.05) between these groups. In contrast, the high-dose group had an average pH of 6.2 ± 0.06, showing a significant difference (*P* < 0.05) compared to both the control and low-dose MP treatment groups (Fig. 5A). Additionally, MP exposure had a significant impact on the glucose levels in extrapallial fluid of oysters (Fig. 5B). Glucose levels appeared to decrease between the control (34.08 ± 0.77 mg/dl) and low-dose (31.4 ± 1.04 mg/dl) treatments (*P* < 0.05, Student *t*-test) and even further decrease between the control and high-dose (31.3 ± 0.69 mg/dl) treatments (*P* < 0.01, Student *t*-test) groups (Fig. 5B).

### 3.4 Effects of MP mixtures on nitrotyrosine protein expression in tissues

**Fig. 6:**
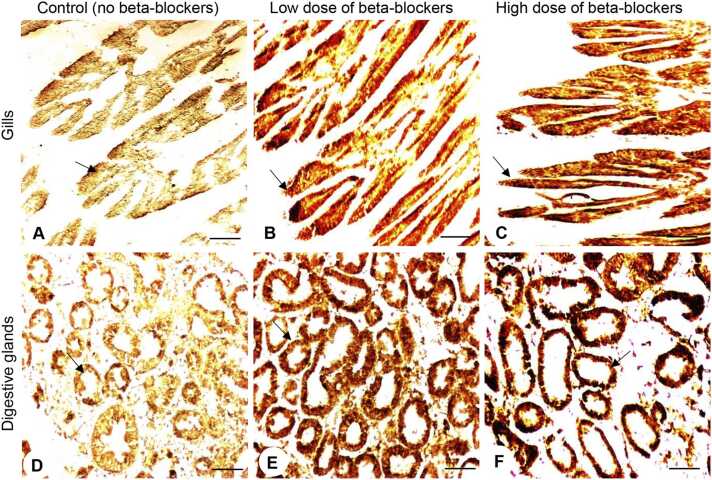
Effects of beta-blockers on 3-nitrotyrosine protein expression in tissues of American oysters. Short-term exposure (one week) to metoprolol and propranolol mixtures on 3-nitrotyrosine protein (NTP) expression in the gills (A-B) and digestive glands (D–F) of oysters. Representative images of gills and digestive glands collected from oysters exposed to control (A, D), low dose (B, E), and high dose (C, F) treatment groups. A darker brown color and arrows denote increased NTP expression in tissues. Scale bar = 100 µm.

**Fig. 7:**
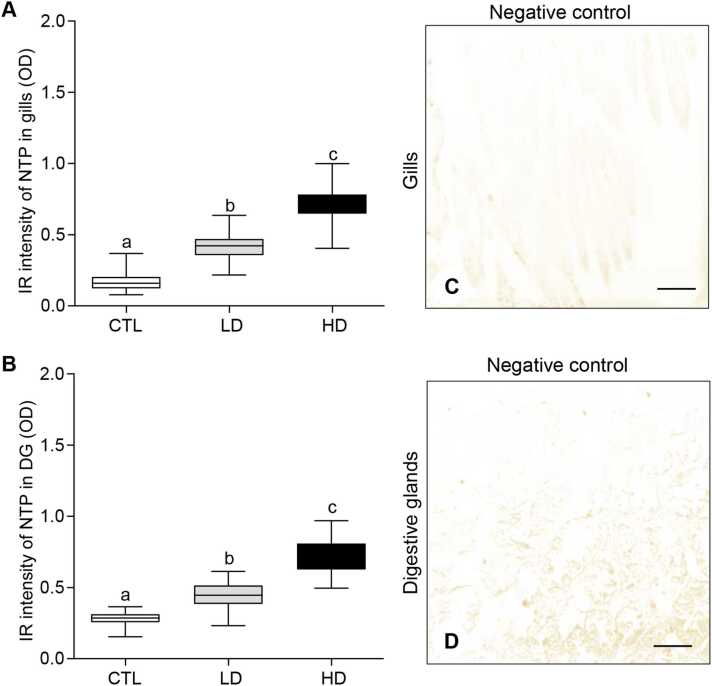
Effects of beta-blockers on 3-nitrotyrosine protein expression in tissues of American oysters. Short-term exposure (one week) to metoprolol and propranolol mixtures on immunoreactive (IR) intensity of 3-nitrotyrosine protein (NTP) in the gills (A) and digestive glands (DG) (B) of oysters. Whiskers indicate the minimum and maximum values for each data set. The center line within the whisker box indicates the mean value (N = 80). Different letters indicate significant differences (one-way ANOVA followed by Tukey’s test, *P* < 0.05). (C, D) Negative controls showing the absence of IR signal of NTP in tissues. Scale bar = 100 µm. CTL, control; LD, low dose; HD, high dose; OD, optical density.

Since 3-nitrotyrosine protein (NTP) serves as a biomarker for nitrative stress in marine bivalves exposed to environmental stressors, we assessed NTP expression in the gills and digestive glands using an immunohistochemical assay (Fig. 6). Oysters exposed to both low- and high-dose of MP mixtures exhibited increased NTP expression in the gills (Fig. 6A–C) and digestive glands (Fig. 6D–F). By analyzing the immunoreactive (IR) intensity, the average optical density (OD) of NTP in the gills of the control group was (OD: 0.17 ± 0.01) (Fig. 7A). The average OD of NTP was 0.42 ± 0.009 in the low-dose MP treatment group, representing an ∼2.5-fold increase compared to the control group. The high-dose MP treatment group showed an average OD of 0.72 ± 0.016, which was 4.2-fold higher than the control group (Fig. 7A). Similarly, the digestive glands exhibited a significant elevation in NTP expression in oysters exposed to the different concentrations of MP mixtures (Figs. 6D–F, 7B). The low-dose MP treatment group showed an average OD of 0.44 ± 0.01, representing a 1.58-fold increase over the control. The high-dose MP treatment group had an average OD of 0.72 ± 0.13, which was significantly higher (*P* < 0.05) around 2.6-fold compared to the control group (Fig. 7B). No IR signal was detected in the gill and digestive gland tissue sections when the primary antibody of NTP was omitted (Fig. 7C, D).

### 3.5 Effects of MP mixtures on acetylcholinesterase expression in tissues

**Fig. 8:**
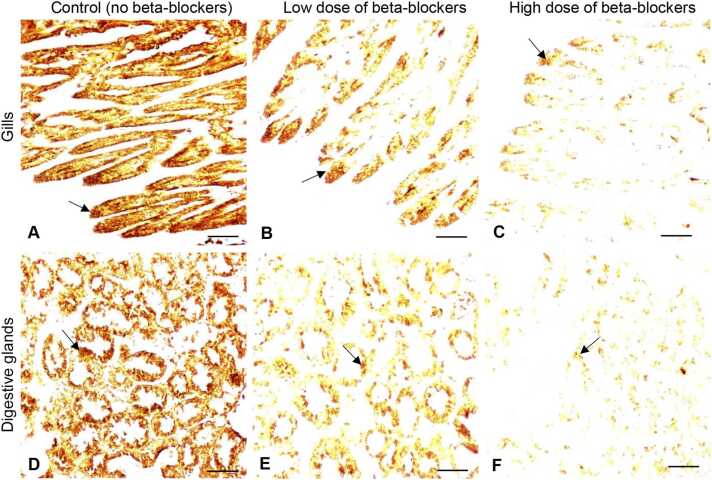
Effects of beta-blockers on acetylcholinesterase expression in tissues of American oysters. Short-term exposure (one week) to metoprolol and propranolol mixtures on acetylcholinesterase (AChE) expression in the gills (A-B) and digestive glands (D–F) of oysters. Representative images of gills and digestive glands collected from oysters exposed to control (A, D), low dose (B, E), and high dose (C, F) treatment groups. A darker brown color and arrows denote increased AChE expression in tissues. Scale bar = 100 µm.

**Fig. 9:**
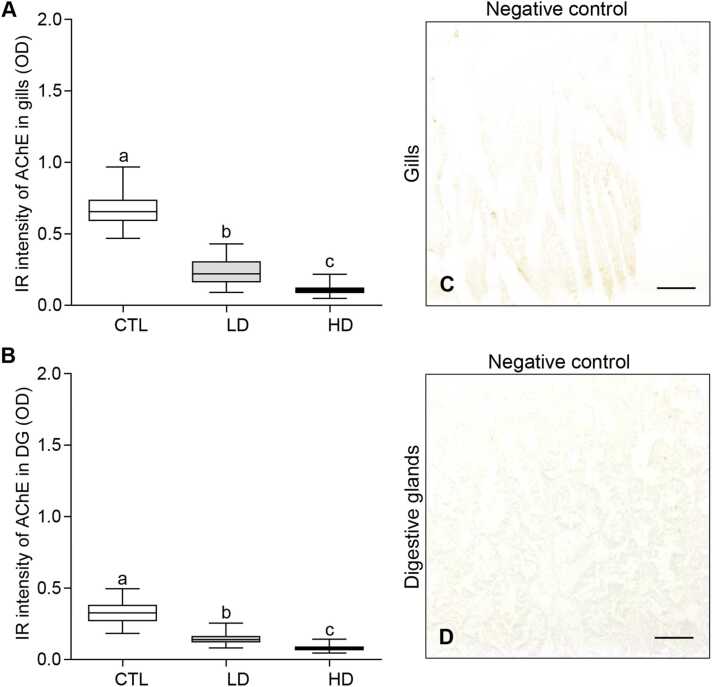
Effects of beta-blockers on acetylcholinesterase expression in tissues of American oysters. Short-term exposure (one week) to metoprolol and propranolol mixtures on immunoreactive (IR) intensity of acetylcholinesterase (AChE) in the gills (A) and digestive glands (DG) (B) of oysters. Whiskers indicate the minimum and maximum values for each data set. The center line within the whisker box indicates the mean value (N = 80–82). Different letters indicate significant differences (one-way ANOVA followed by Tukey’s test, *P* < 0.05). (C, D) Negative controls showing the absence of IR signal of AChE in tissues. Scale bar = 100 µm. CTL, control; LD, low dose; HD, high dose; OD, optical density.

Oysters exposed to MP mixtures displayed a substantial decrease in acetylcholinesterase (AChE) expression in both the gills (Fig. 8A-C) and digestive glands (Fig. 8D-F). In the gill tissues, the IR intensity of AChE expression decreased significantly (*P* < 0.05, Tukey’s test) with increasing doses of MP mixtures. The low-dose treatment group showed an average OD of 0.23 ± 0.009, representing a 2.9-fold reduction compared to the control group (Fig. 9A). In the high-dose group, the average OD was 0.11 ± 0.003, which was ∼6-fold significant (*P* < 0.05) lower than the control group (Fig. 9B). Likewise, the expression and IR intensity of AChE in the digestive glands significantly decreased (*P* < 0.05) as dosages increased (Figs. 8D–F, 9B). The low-dose MP treatment group showed a reduced expression of AChE (∼2.26-fold) in the digestive glands. Similarly, the high-dose treatment group exhibited an even lower reduction of AChE expression (∼4.2-fold) compared to the control group (Fig. 9B). No IR signal was detected in the gill and digestive gland tissue sections when the primary antibody of AChE was omitted (Fig. 9C, D).

**Fig. 10:**
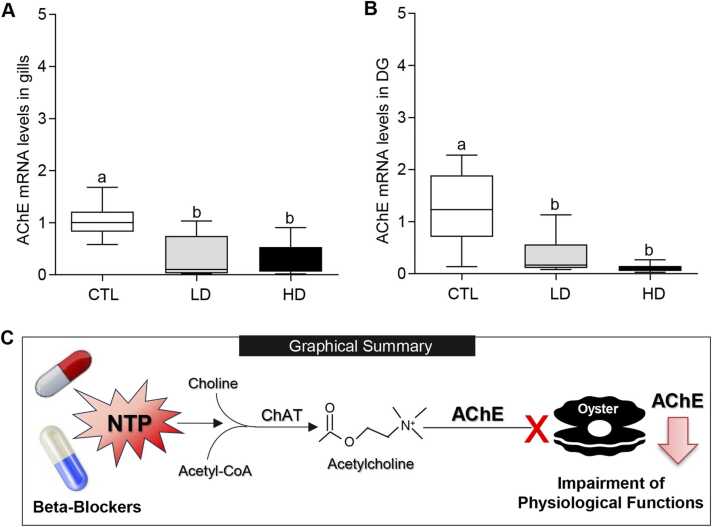
Effects of beta-blockers on acetylcholinesterase mRNA levels in tissues of American oysters. (A, B) Short-term exposure (one week) to metoprolol and propranolol mixtures on acetylcholinesterase (AChE) mRNA levels in the gills (A) and digestive glands (B) of oysters. Whiskers indicate the minimum and maximum values for each data set. The center line within the whisker box indicates the mean value (N = 8). Different letters indicate significant differences (one-way ANOVA followed by Tukey’s test, *P* < 0.05). (C) Graphical summary of upregulation of 3-nitrotyrosine protein (NTP) and downregulation of AChE and impairment of physiological functions of oysters after exposure to beta-blockers. ChAT, choline acetyltransferase; CTL, control; LD, low dose; HD, high dose.

Because AChE is known to play important physiological roles, we further assayed the mRNA expression pattern of AChE in oyster tissues using quantitative real-time PCR (qRT-PCR). qRT-PCR results showed that AChE mRNA levels were significantly decreased (*P* < 0.05, Tukey’s test) in the gills and digestive glands of oysters after exposure to low- and high-dose MP treatment groups compared to the controls (Fig. 10).

## 4 Discussion

Pollutants and contaminants have diverse and often detrimental effects on aquatic ecosystems. Among these, pharmaceutical contaminants, such as beta-blockers like propranolol and metoprolol (MP), have been shown to disrupt normal physiological functions in aquatic organisms [11], [47], [48]. These contaminants can impair growth, alter behavior, and hinder reproduction in aquatic species [49], [50]. Moreover, beta-blockers interfere with hormonal regulation and stress response systems, crucial processes for maintaining homeostasis in aquatic organisms [51]. Disruption in these systems may lead to ecological consequences, such as population declines and impaired ecosystem health and aquaculture sustainability. For these reasons, the potential impacts of pharmaceuticals on the ecosystem health and ecological consequences of oysters including other non-target aquatic organisms (e.g., fish, shrimp, clamps, etc.) are of significant interest. However, studies on the physiological effects of beta-blockers like MP on marine mollusks are still limited. Our analysis revealed that short-term (one week) exposure to environmentally relevant concentrations of MP significantly impacted the gills and digestive glands of American oysters (*Crassostrea virginica*). At low (250 ng/L metoprolol, 50 ng/L propranolol) and high doses (650 ng/L metoprolol, 250 ng/L propranolol) of MP exposure caused notable changes in tissue architectures and body fluid conditions. It also triggered nitrative stress (i.e., 3-nitrotyrosine protein, NTP; a biomarker of protein nitration) and led to a significant decrease in acetylcholinesterase (AChE, a key biomarker in nervous system) expression. Histological analysis revealed that exposure to MP mixtures resulted in significant damage to the gill filaments, leading to structural degradation of oysters. The area of lumen in the digestive glands exhibited notable decay, while the number and distribution of hemocytes were altered, indicating a disruption in the immune response. Additionally, there was an enlargement of blood sinuses within the connective tissue, suggesting impaired tissue integrity. Biochemical analysis revealed a dose-dependent decrease in extrapallial (EP) fluid glucose levels following exposure to MP. As the concentrations of these beta-blockers increased, glucose levels dropped significantly. Additionally, exposure to MP mixtures influenced extrapallial fluid pH, indicating that both metabolic and acid-base balance were disrupted. Immunohistochemical assay revealed a dose-dependent (i.e., low-dose versus high-dose beta-blockers) increase in NTP expression in the gills and digestive glands of oysters exposed to MP mixtures, while AChE expression was decreased in tissues. These results highlight the significant impact of MP mixture on tissue architecture and extrapallial fluid conditions, leading to disruption in oysters' energy metabolism, compromised immune response, and impaired biochemical and physiological homeostasis.

### 4.1 Effects of MP mixture on tissue architecture in oysters

The filter-feeding behavior of bivalves exposes them to environmental pollutants, making it easier to detect and measure these contaminants in their tissues [52], [53]. Studies have shown that various environmental pollutants have significant impacts on oyster tissues. For instance, Gan et al. [54] highlighted that polycyclic aromatic hydrocarbon (PAH, an organic compound) exposure leads to substantial histopathological changes in the gills of oysters, compromising their respiratory and filtration functions. Similarly, Suvetha et al. [55] demonstrated that exposure to cypermethrin, a synthetic pyrethroid insecticide, significantly induces oxidative stress, which further impairs osmoregulatory processes in the gills of teleost fish. Our study identified significant damage to the gills of oysters following short-term (one week) exposure to the beta-blockers, MP mixtures. Exposure to MP mixture resulted in expansion of the gill lamellae and intra-lamellar spaces in oysters as well as displaying disruption of epithelial cells in the gills and damage to the gill cilia and lamellae. Recently, Dong et al. [56] demonstrated that exposure to cadmium significantly induces oxidative stress in the gills, alters tissue structure, and disrupts energy metabolism in the Pacific oyster (*Crassostrea gigas*). These findings highlight the vulnerability of gill tissues to a variety of environmental pollutants, from heavy metals and pesticides to pharmaceuticals like beta-blockers, which can lead to structural damage, induced oxidative stress, and impaired physiological functions in fish and shellfish species.

An important finding in our study is that the MP mixture disrupts the tissue architecture of digestive glands in oysters. Notably, the epithelial tubules of the digestive glands became radically enlarged and lost their natural structure, exhibiting distortion and enlargement ∼7.2-fold in high-dose MP treatment groups. Edge et al. [57] conducted a study across several estuaries in Australia and demonstrated that heavy metal contamination from environmental sources like copper (10, 50, and 100 μg/L for 96 h) led to severe cell damage in the digestive glands of Sydney rock oyster (*Saccostrea glomerata*). Recently, Chowdhury and Rahman [58] evaluated the impact of Roundup® exposure (low dose: 0.5 µg/L, high dose: 5 µg/L for one week) in the digestive glands of American oysters. The results showed significant morphological alterations, including cellular degeneration and oxidative stress, which compromised critical digestive functions in bivalve mollusks. The study underlines the environmental hazards associated with Roundup, a widely used agricultural pesticide, demonstrating its potential to cause cellular damage even at low concentrations. Another study by Nobre et al. [59] discovered that microplastics associated with a common pharmaceutical pollutant, triclosan, led to cellular damage and impaired enzymatic function in the digestive glands (250 mg/L of microplastics spiked with triclosan for 7 days) of Brazilian oysters (*Crassostrea brasiliana*). Taken together, it has been postulated that exposure to environmental contaminants significantly disrupts the tissue architecture of aquatic organisms, inducing notable morphological alterations in both the gills and digestive glands of oysters, agreeing with our present study.

### 4.2 Effects of MP mixture on mucous secretion in tissues

Mucous serves as a multipurpose tool in oysters, offering multiple protective and functional purposes. In gills, mucus helps trap suspended particles and contaminants from the water, facilitating filtration and protecting delicate gill tissues from damage caused by environmental pollutants [54]. In addition, mucus serves a protective role by encapsulating ingested particles, including food and contaminants in the digestive glands, aiding in their safe transport through the digestive tract. It also plays a key role in neutralizing harmful substances and reducing the impact of toxins on digestive tissues in marine mollusks [60], [61]. The mucus secreted in the gills helps capture and suspend food particles from surrounding water. It also plays a critical role in the feeding process of oysters [61]. The suspended food is then moved along the gill surface in a layer of mucus facilitating efficient filtration. Once captured, the food is transported to the digestive glands where mucus continues to play a role by coating the food and aiding in its movement through the digestive tract [61]. Our study demonstrates that exposure to MP mixtures leads to a substantial reduction in mucous secretion in both the gill lamellae (approximately 8.8-fold) and the vacuoles of the digestive glands (∼4.7-fold). There are mixed results regarding mucous secretion increasing or decreasing in various studies. For example, Costa et al. [62] showed that exposure to biofloc systems with elevated levels of suspended solids (low concentration: 100 mg/L, medium concentration: 100–200 mg/L, high concentration: 200 + mg/L for 28 days) led to significant physiological responses, including increased mucus production to manage the higher organic load in mangrove oysters (*Crassostrea gasar*). Recently, Stara et al. [63] demonstrated that exposing Mediterranean mussels (*Mytilus galloprovincialis*) to a neonicotinoid insecticide (7.77 mg/L for 20 days) increased mucus secretion and caused inflammation in the gills. Meanwhile, Khan et al. [64] found a loss of mucous cells and mucous production in graphene oxide exposed (low concentration: 1 mg/L, high concentration: 10 mg/L for 72 hours) to American oysters. Similarly, Ahmed and Rahman [42] found a decrease in mucus production in the gills and digestive glands of oysters exposed to a pesticide mixture (low concentration: 0.4 μg/L of atrazine, 0.5 μg/L of Roundup, and 0.5 μg/L of 2,4-D; and (high concentration: 0.8 μg/L of atrazine, 1 μg/L of Roundup, and 1 μg/L of 2,4-D for one week). Exposure to contaminants has been documented to alter mucus secretion in oysters, regardless of whether it increases or decreases. Our findings indicate that reduced mucus secretion in the gills and digestive glands, resulting from exposure to beta-blockers, can severely impair key physiological functions in oysters. Collectively, this reduction leads to compromised digestion, osmoregulation, and hindered food transport in bivalve mollusks.

### 4.3 Effects of MP mixture on hemocyte aggregation in connective tissues

Hemocytes are the primary immune cells in bivalves responsible for handling foreign particles and pathogens [65]. Hemocytes are also involved in performing critical functions in oysters such as phagocytosis, wound healing, nutrient transport, and shell formation [66]. An increase in hemocyte density indicates a heightened immune response, often due to the presence of pathogens, environmental stress, or tissue injury [67]. Auffret and Oubella [68] observed hemocyte aggregation in the Pacific oysters exposed to tributyltin (TBT; low dose: 50 ng/L, high dose: 500 ng/L) *in vitro*. Chowdhury and Rahman [58] observed that exposure to environmentally relevant dosage of Roundup paired with heat stress (low dose: 0.5 µg/L, high dose: 5 µg/L for one week) and (22 °C to 30 °C within 7 days) spontaneous hemocyte aggregation and led to the enlargement of blood sinuses in the connective tissues of oysters *in vivo*. Similarly, Luo and Wang [69] observed an increased number of granulocytes after exposure to zinc (50 μg/L and 300 μg/L for 14 days), indicating the increase of hemocyte-mediated immunity in the Hong Kong oysters (*Crassostrea hongkongenesis*). Moreover, Ahmed and Rahman [42] reported significant alterations in hemocyte density in the American oysters exposed to pesticides mixture (low concentration: 0.4 μg/L atrazine, 0.5 μg/L Roundup, and 0.5 μg/L 2,4-D; high concentration: 0.8 μg/L atrazine, 1 μg/L Roundup, and 1 μg/L 2,4-D for one week). Our research aligns with these findings, demonstrating a dose-dependent MP mixture increase in hemocyte aggregation in oysters, signaling a compromised immune response.

### 4.4 Effects of MP mixtures on extrapallial fluid conditions

Oyster extrapallial fluid (also called body fluid) is a fluid located in the extrapallial space, which is the gap between the mantle and the inner surface of the shell in bivalves [60], [70]. Extrapallial fluid plays an essential role in the biomineralization process of oyster shells, contributing to both shell formation and maintenance [71]. It is rich in key components such as amino acids, carbohydrates, lipids, glycoproteins, and proteins. Along with high concentrations of calcium and bicarbonate ions which are critical for producing calcium carbonate (CaCO₃), the primary mineral that constitutes the oyster’s shell [60], [72]. The extrapallial fluid also helps maintain the ionic balance necessary for shell deposition, acting as a buffer to prevent rapid changes in pH that could disrupt shell formation [73]. Additionally, extrapallial fluid supports immune functions by participating in the isolation of foreign particles or pathogens, assisting in the oyster's defense mechanisms [74]. Extrapallial fluid pH is an important marker on the impacts of environmental stressors on oysters, when oysters are exposed to contaminants such as heavy metals or pollutants, the pH of the EP fluid can decrease, leading to disruptions in shell formation [39]. Boitel and Truchot [75] discovered a significant decrease in extrapallial fluid pH level (7.8–7.45) in the shore crab (*Carcinus manenas*) when exposed to Cu (0.5, 1, and 2 mg/L for 20 days). Similarly, in our study we measured oyster extrapallial fluid pH levels, finding a significant decrease between the high-dose and control/low-dose MP treatment groups.

In addition to extrapallial pH, glucose is one of the primary carbohydrates found in the extrapallial fluid of oysters, serving as an immediate source of energy for function and immune responses, also being directly influenced by external and internal factors such as heat stress or contaminants [76], [77]. Since glucose in the extrapallial fluid is responsive to a bivalve’s surroundings, this makes it a reliable indicator of physiological health, environmental stress, and water quality [78], [79]. Caldari-Torres et al. [76] found that stressed crayfish (genus *Orconestes*) displayed higher glucose levels of 45 ± 14.3 mg/dl when compared to the control group (28.4 ± 8.8 mg/dl). Billah and Rahman [78] demonstrated that oysters exposed to one week of heat stress (28 and 32 °C) displayed elevated glucose levels respective to amount of high temperature. Brew et al. [80] found that exposure to pharmaceuticals (1 µg/L for 10 days) and personal care products, including fluoxetine, diphenhydramine, *N,N*-diethyl-meta-toluamide, and 17α-ethynylestradiol, led to increased glucose levels in the extrapallial fluid of American oysters. Rahman et al. [79] also reported an increase in glucose levels (∼1.9-fold) in oysters subjected to heat stress (28 and 32 °C for one week). In our study, we discovered a significant decrease in glucose levels (around 1.01- and 1.09-fold) of oysters for low- and high-dose MP treatment groups suggesting that environmental contaminants and/or heat stress directly alter glucose levels in the extrapallial fluid of bivalve mollusks. These fluctuations of extrapallial fluid glucose affect growth, development, reproduction, and immune function, as well as overall survival in oysters during environmental stress and/or contaminants.

### 4.5 Effects of MP mixtures on NTP expression in tissues

Nitrative stress in mollusks, including bivalves, is often a result of environmental contaminants such as heavy metals and pollutants, which cause an imbalance between RNS and the organism's ability to detoxify them. This type of stress can result in oxidative/nitrative damage to DNA, lipids, and proteins [81], [83]. In our study, we measured NTP, a well-established biomarker of protein nitration [82], expression in the gills and digestive glands of oysters. We found a significant amplification in NTP expression in gills (∼4.2-fold) and digestive glands (∼2.6-fold) of oysters respective to MP treatment groups. These findings coincide with Dash and Rahman [39] who observed an upregulation in NTP in oysters subjected to TBT (0.1 and 1 μg/L for one week). Chowdhury and Rahman [58] recorded an upregulation in NTP in the gills and digestive glands of oysters exposed to Roundup (low dose: 0.5 µg/L, high dose: 5 µg/L for one week) and heat stress (22 °C to 30 °C for seven days. Nash et al. [43] also observed an upregulation in NTP and NOx levels in the gonadal tissues of oysters when subjected to heat stress for one week (28 and 30 °C). Our study corresponds to these previous studies suggesting that environmental contaminants such as pesticides, anti-fouling agents (i.e., TBT), and even heat stress directly cause an increase in protein nitration (i.e, NTP) which may lead to decreased cholinergic enzyme (e.g., AChE) activity in tissues of oysters.

### 4.6 Effects of MP mixtures on AChE expression in tissues

AChE is extensively utilized as a biomarker to assess the neurotoxic effects of environmental pollutants in aquatic organisms like fish and shellfish [84], [85]. An important finding of this study is that exposure to MP mixture causes marked declines in AChE expression and mRNA levels in the gills and digestive glands of oysters. This interference in AChE expression may affect normal functions of cholinergic enzyme, leading to physiological imbalances, and diminished growth of oysters [86]. Similarly, Haque et al. [87] observed that exposure to the antifouling agent chlorothalonil (0.1, 1, and 10 μg/L for 96 h) significantly reduced AChE activity in the gills of blue mussels (*Mytilus edulis*) and Pacific oysters. Bernal-Hernandez et al. [34] observed that Cortez oysters (*Crassostrea corteziensis*) exposed to the pesticide dichlorvos (1 mg/L for 6 h) displayed a 65 % decline in AChE activity in the adductor muscles, gills, and mantles. Park et al. [86] observed that Pacific oysters exposed to TBT (0.01, 0.1, and 0.01 μg/L for 96 h) showed a significant reduction in AChE activity in the gills. Recently, Dash and Rahman [39] observed oysters exposed to TBT (0.1 μg/L and 1 μg/L for 1 week) displayed lower AChE expression in the gills and digestive glands. Collectively these findings concur with our results and suggest that environmental contaminants disrupt AChE activity in tissues of bivalve mollusks, which could potentially affect critical processes such as deeding behavior, impair physiological functions, stress responses, and inevitability leading to an increase in mortality rates.

### 4.7 Limitation and future studies

While this study demonstrates the acute exposure to beta-blockers mixture on oysters, the short-term (one week) exposure and controlled laboratory conditions limit insights into chronic or ecologically realistic impacts. Additional research is necessary to prioritize long-term and field-based studies of beta-blockers to assess bioaccumulation kinetics, multigenerational effects, and interactions with other environmental stressors such as elevated temperature [45], high salinity stress [88], coexisting pollutants [42], [56], etc. in aquatic organisms.

## 5 Conclusion

Pharmaceutical drugs, such as metoprolol and propranolol, commonly prescribed for the treatment of cardiovascular conditions in humans, have been increasingly detected in coastal ecosystems, where they can cause severe tissue damage in marine organisms. Notably, the accumulation of these beta-blockers in aquatic environments is an emerging driver of biodiversity decline, alongside other chemical contaminants. Although developed for human health applications, these substances can adversely affect non-target organisms (e.g., fish and shellfish species), disrupting critical physiological functions and threatening the health and sustainability of vulnerable marine populations. This study provides the first clear evidence that short-term exposure to the beta-blocker mixture (metoprolol and propranolol) alters tissue architecture, EF fluid conditions, and nitrative stress protein (i.e., NTP) and cholinergic enzyme (i.e., AChE) expressions in tissues of oysters. The finding that beta-blockers drastically increase NTP and decrease AChE expressions in tissues suggests that exposure to pharmaceutical pollution can hamper osmoregulation, weaken the immune system, impair reproductive function, and negatively affect growth in oysters (Fig. 10C). Moreover, pharmaceuticals should be disposed of properly to prevent their release into aquatic environments. As pharmaceutical contamination continues to increase, marine bivalves and other sensitive species are likely to experience even greater physiological stress.

## Funding

This study was funded in part by the College of Sciences, UTRGV SEED grant (grant no. 31050258 to Dr. Md Saydur Rahman).

## CRediT author contribution statement

Andrew Salinas conducted experiments, collected data, analyzed, and wrote and edited the article. Md Saydur Rahman conceived the research idea, supervised the experiments, and edited the article. The authors have read and agreed to publish the final version of the article.

## CRediT authorship contribution statement

**Md Saydur Rahman:** Writing – review & editing, Visualization, Validation, Supervision, Software, Resources, Project administration, Methodology, Investigation, Funding acquisition, Formal analysis, Data curation, Conceptualization. **Andrew Salinas:** Writing – original draft, Visualization, Validation, Methodology, Investigation, Formal analysis, Data curation, Conceptualization, Writing – review & editing.

## Declaration of Competing Interest

The authors declare that they have no known competing financial interests or personal relationships that could have appeared to influence the work reported in this paper.

## Data Availability

Data will be made available on request.
